# Paradoxical experiences of healthcare workers during COVID-19: a qualitative analysis of anonymous, web-based, audio narratives

**DOI:** 10.1080/17482631.2023.2184034

**Published:** 2023-03-02

**Authors:** Lori Lackman Zeman, Sujoy Roy, Pranjali P. Surnis, Jason Adam Wasserman, Kathleen Duchak, Ramin Homayouni, Elie Mulhem

**Affiliations:** a Department of Family Medicine, Beaumont Health, Troy, MI, USA; b Department of Family Medicine and Community Health, Oakland University William Beaumont School of Medicine, Rochester, MI, USA; c Department of Foundational Medical Studies, Oakland University William Beaumont School of Medicine, Rochester, MI, USA; d Department of Computer Science and Engineering, Oakland University, Rochester, MI, USA

**Keywords:** COVID-19, healthcare workers, paradoxical experiences, social isolation, social connectedness, distress, meaningfulness, qualitative, web application, audio narratives

## Abstract

**Purpose:**

To gain a deeper understanding of healthcare workers experiences during COVID-19 using an anonymous, web-based, audio narrative platform.

**Methods:**

Data were collected from healthcare workers in the midwestern United States using a web-enabled audio diary approach. Participant recordings were analysed using a narrative coding and conceptualization process derived from grounded theory coding techniques.

**Results:**

Fifteen healthcare workers, in direct patient care or non-patient care roles, submitted 18 audio narratives. Two paradoxical themes emerged: 1) A paradox of distress and meaningfulness, where a harsh work environment resulted in psychological distress while simultaneously resulting in new rewarding experiences, sense of purpose and positive outlooks. 2) A paradox of social isolation and connection, where despite extreme isolation, healthcare workers formed intense and meaningful interpersonal connections with patients and colleagues in new ways.

**Conclusions:**

A web-enabled audio diary approach provided an opportunity for healthcare workers to reflect deeper on their experiences without investigator influence, which led to some unique findings. Paradoxically, amid social isolation and extreme distress, a sense of value, meaning and rewarding human connections emerged. These findings suggest that interventions addressing healthcare worker burnout and distress might be enhanced by leveraging naturally occurring positive experiences as much as mitigating negative ones.

## Introduction

The Coronavirus Disease 2019 (COVID-19) pandemic is unprecedented in the contemporary era, both in its global scale and as a collective societal experience (Giannopoulou et al., 2021; Patterson et al., 2021; Tsamakis et al., 2021). As of October 2022, there have been more than 625 million confirmed cases of COVID-19, including over 6.5 million deaths world-wide reported to the World Health Organization (WHO), leaving essentially no country untouched (WHO Coronavirus COVID, 2022). In contrast, in 2003 a total of 8,098 probable cases of SARS was reported to the WHO with 774 SARS-related deaths, touching 29 countries (Surveillance Case Definition for Severe Acute Respiratory Syndrome SARS and Update on SARS Cases — United States and Worldwide, 2003). This pandemic put unprecedented pressure on the world’s healthcare systems and on healthcare workers who faced unusual levels of stress while attempting to address the virus and its devastating consequences (De Kock et al., 2021). Evidence-based qualitative techniques can be highly useful for examining healthcare environments during unprecedented and uncertain times to capture a deeper understanding of healthcare workers experiences (Foley & Timonen, 2015; Popay & Ma, 1998; Sofaer, 1999).

Qualitative studies from around the world reported on healthcare workers experiences during the COVID-19 pandemic, and documented their struggles and triumphs (Al-Ghunaim et al., 2021; Hoernke et al., 2021; Marsaa et al., 2021; Parsons Leigh et al., 2021; Q. Liu et al., 2020; Y. E. Liu et al., 2020; Yau et al., 2021). A systematic review of 28 qualitative studies examining the psychosocial impact of the pandemic on nurses revealed 3 main theses (Xu et al., 2021). The most common emotional reactions reported were fear of infection and facing many uncertainties during the pandemic. Second, nurses reported negative psychological and physical impacts of changing work environments, frequent changing of care guidelines and witnessing the suffering and death of patients. Third, nurses used internal and external support for coping strategies, they relied on family members and friends for emotional support, expressing a sense of satisfaction and pride in their contributions to disease control. In this systematic review, most studies collected data through interviews, whereas fewer used surveys or questionnaires, and one study used anonymous digital audio recordings.

The use of qualitative audio diaries originated in the field of psychology and has expanded into other disciplines (Crozier & Cassell, 2016; van Eerde et al., 2005; Verma, 2021). Audio diaries offer many advantages over other qualitative data collection techniques, particularly in periods of change or stress (Crozier & Cassell, 2016; Verma, 2021). Because audio diaries can be unprompted, anonymous, and completed at any time and place convenient to the participant they offer many advantages over other forms of qualitative data collection. Some of these advantages include: 1) Immediacy and accuracy of data capture (Monrouxe, 2009), 2) providing more depth and reflection of experiences (Markham & Couldry, 2007), 3) lower attrition due to ease of use (Markham & Couldry, 2007), 4) minimizing the influence of the researchers on the participant’s responses (Monrouxe, 2009), and 5) added benefit of sense-making, alleviating discomfort from the experience, and facilitating coping with stressors (Pennebaker & Seagal, 1999).

Due to the many potential advantages of audio diaries, we developed an anonymous, web-based, audio-recording platform to document the experiences of healthcare workers during the first surge of the COVID-19 pandemic. This platform provided flexibility for the participants to privately share their thoughts at any time that was convenient for them. We hypothesized that this approach would result in unique findings leading to an increased understanding of how healthcare workers were affected and insights to better prepare healthcare workers for the future.

## Methods

### Setting and study population

**Figure 1.**
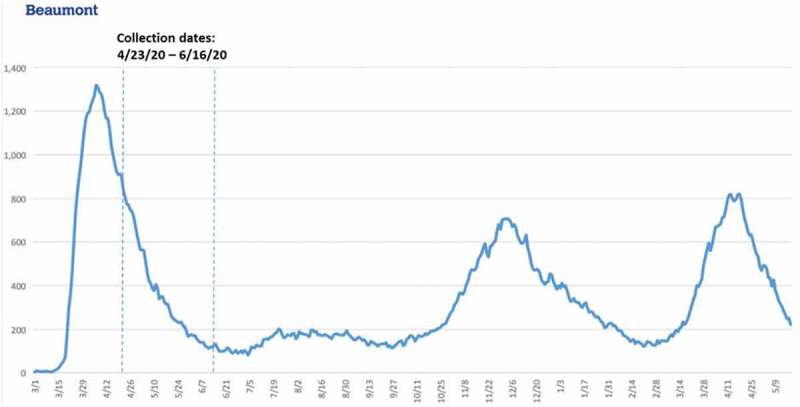
Number of hospitalized COVID-19 patients at study health system during the first year of the pandemic. the dashed lines indicate the period when the audio recordings were collected.

This study took place in the midwestern United States. Participants were recruited from a large health system consisting of eight hospitals and multiple supporting outpatient facilities. Data reported here represents the first phase of a two-phase study. Data collection in this phase took place between April 23 and 16 June 2020, during the decline of the first local COVID-19 surge, as measured by the number of patients hospitalized with COVID-19 across the study healthcare system (Figure 1). Because COVID-19 put a unique strain on the entire healthcare system, we wanted to give voice to all our healthcare workers to learn more about how workers in different roles were impacted. Thus, recruitment targeted all healthcare workers, not just those on the frontline or involved in direct patient care. We used a convenience sampling approach. Invitations to participate were periodically included in daily COVID-19 update emails sent out by the health system’s communication department to all employees and contracted providers.

### Data collection

**Figure 2.**
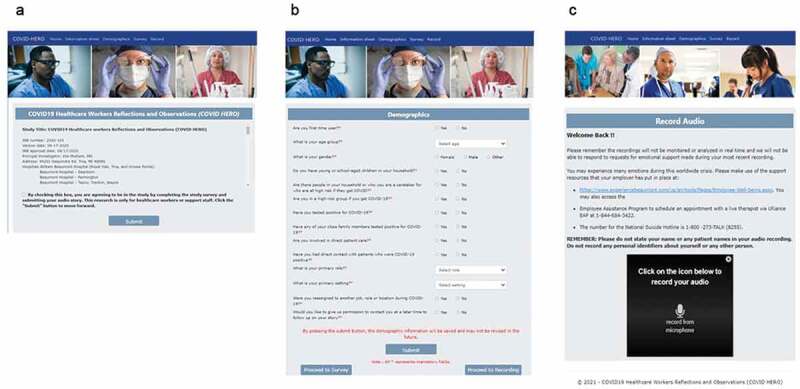
Screenshots of the webpages corresponding to the consent, demographic, and audio recording features. the homepage (a) of the website displays the entire consent form, which must be accepted before proceeding to the following pages. In addition, the demographics questionnaire (b) must be completed and submitted before the user is able to proceed to the audio recording page (c). Returning users who had successfully consented and submitted a demographics form would proceed directly to the recording page in subsequent visits.

The recruitment announcements directed interested participants to a website (COVID19HERO.health), where they could consent to participate in the study, provide basic demographic information, and submit an audio recording (Figure 2). In the consent form, participants were told the following: *You can talk about whatever you would like to share. This might include stories about your own experience, stress or emotional response; observations regarding the health system or individual preparedness, responsiveness, or adaptation regarding the pandemic; or anything regarding individual or collective ability to care for our patients and ourselves*. There were no specific prompts once they completed the demographic data collection and proceeded to the recording page. A list of support resources was provided in case participants were in distress. Participants were asked to not include any personal or patient identifiers in their recordings. The audio recording was limited to 5 minutes. Participants had the option to return to the website and record as often as they desired.

### Web application development

The web application was developed in a.NET framework. The application was deployed on a secure HTTPS server in the Amazon Web Services (AWS) environment. The audio recordings were enabled using a JavaScript plugin provided by a GDPR and HIPAA compliant third-party subscription service called “CameraTag.” A schematic diagram of the system architecture and data flow is shown in Supplemental Figure 1. All collected information was securely transmitted and stored in an AWS Relational Database Service (RDS) and AWS S3 buckets in encrypted format. Additionally, the device information was collected via browser cookies to keep track of unique participants and to match the demographic information to multiple recordings.

### Data analysis

Audio recordings were transcribed using the closed caption transcription service provided by CameraTag. One of the study investigators (KC) listened to the recordings and corrected any transcription errors. The transcribed data were analysed using a narrative coding and conceptualization process. Though this study does not employ Grounded Theory (GT) proper, the analytic process is derived from the coding and conceptualization processes of GT (Charmaz, 2014; Glaser & Strauss, 2019). One of the investigators (J.W.), an expert in qualitative research and grounded theory, provided training and oversight of this process. We followed these steps in our data analysis, mimicking the steps taken in GT coding: Four investigators (L.Z, R.H, K.C. and E.M) independently coded transcripts line-by-line using an open-coding technique. Memos were recorded by each coder during the coding process to track potential emergent themes. Once this was completed, coders met to create a combined master list of codes by synthesizing their independent codes into amalgams that more robustly captured what was emergent in the data. While there was some variance across coders initially, it was primarily due to the degree to which some coders combined codes into fewer codes at this step. Differences were resolved through discussion and mutual consensus was obtained. Investigators then independently raised the agreed upon open-codes into axial codes by bringing similar or related codes together under aggregate constructs. The team then met to consolidate independently derived axial codes into categories using a similar process of synthesis as above to create the strongest aggregate expressions of concepts in the data. Finally, the team activated categories and/or connected multiple categories together into propositions that captured the larger emergent themes of the data.

### Patient and public involvement

Patients and/or the public were not involved in the design, conduct, reporting, or dissemination plans of this research.

## Results

During the study period, 127 participants consented to participate in the study and completed the demographic form. A set of 15 unique participants submitted 18 audio recordings, ranging in length from 39 seconds (69 transcribed words) to the maximum allowed 5 min (1,130 transcribed words). One participant left 2 back-to-back recordings and another left 3 recordings spaced out over 5 days. All recordings were completed between 1:50 am and 12:51 pm, with four occurring between 1:50 am and 4:20 am and three occurring between 6:21 am and 7:52 am.

Table 1 shows the characteristics of participants who completed demographic questions and those who also completed an audio recording. Most participants (60.0%) were between ages 45–64 years, female (86.7%), and involved in direct patient care (66.7%). The largest job category of participants was nurses (26.7%).

**Table I.** Characteristics of participants.

		Completed Demographics		Completed Recording	
		Count	%	Count	%
Number of participants		127		15	
Age					
	18–24 years	12	9.0%	1	6.7%
	25–34 years	31	24.0%	3	20.0%
	35–44 years	24	19.0%	2	13.3%
	45–54 years	36	28.0%	4	26.7%
	55–64 years	21	17.0%	5	33.3%
	65–74 years	3	2.0%	0	0.0%
Gender					
	Male	24	19.0%	2	13.3%
	Female	100	79.0%	13	86.7%
	Gender Other	3	2.0%	0	
Involved in Direct Patient Care		93	73.0%	10	66.7%
Role					
	Nurse	35	28.0%	4	26.7%
	Medical provider	15	12.0%	2	13.3%
	Medical Assistant	18	14.0%	2	13.3%
	Clinical Support	3	2.0%	0	0.0%
	Clerical	6	5.0%	2	13.3%
	Administrative Support	5	4.0%	1	6.7%
	Administrator/manager	12	9.0%	2	13.3%
	Other roles	15	12.0%	1	6.7%
	Other health care provider (e.g., PT/OT, behavioral health, pharmacist)	18	14.0%	1	6.7%

The qualitative analysis resulted in identification of 2 paradoxical themes (Table 2). The paradox of distress and meaningfulness (Theme 1) centred on the physical and psychological demands of a harsh work environment, juxtaposed against work many found incredibly important, meaningful, and rewarding. Similarly, the paradox of isolation and social connection (Theme 2) was manifest in axial codes and categories centred on the required social distancing that healthcare workers had to maintain, while the simultaneous experience of human connection manifested in intense experiences with isolated patients, as well as being “in the trenches” with co-workers calling for increased teamwork in unprecedented ways. Of note, the paradoxical themes occurred both across and within individual stories, emerged in early recordings when COVID-19 numbers were still high as well as towards the end of the surge, and occurred amongst both frontline and non-frontline workers. Approximately 2/3 of our participants described paradoxical experiences. The themes and their facets are elaborated below, with representative quotations.

**Table II.** Major themes and categories summarizing healthcare workers’ experiences.

Theme 1: Harsh work environment resulted in psychological distress, contrasting with personally rewarding experiences and positive outlooks
Categories and axial codes:
Harsh work environment o Surreal, extremely difficult o Uncertainty due to new roles and lack of preparedness o Limited PPE and access to testing o Risk of contamination to self/others o Working with very ill/dying patients o Disruption to regular patient care, negative economic impact, staff loss Psychological distress o Traumatic experience o Fear/anxiety/worry/depression/anger o Sleep/appetite disturbance Rewarding experiences o Pride, sense of duty and meaningful contributions o Positive outlook and hope for the future
Theme 2: Despite extreme isolation, healthcare workers formed intense and meaningful interpersonal connections in new ways.
PPE, redeployment and physical barriers resulted in social isolation and disrupted communication o Isolation from co-workers o Isolation from family o Isolation from patients o Separation between patients and families Intense and meaningful interpersonal interactions emerged o Involvement in intimate patient interactions o Involvement in creating new connections between patients and their families. o Enhanced teamwork

### Theme 1: the harsh work environment resulted in psychological distress, contrasting with personally rewarding experiences and a positive outlook

Nearly all our participants described frightening and traumatic work environments, noting that this was unlike anything they had experienced before or could have imagined. Several described their work settings as “surreal”, while others used words like “nightmare”, “terrifying”, “unimaginable”, “shocking” and compared it to a “war zone”. As an experienced nurse described, “COVID-19 has been the scariest, most challenging nursing experience that I’ve ever had. I’ve been a nurse for almost 31 years and I’m telling you, it is hard.” A medical assistant reflected on the pervasiveness of the feeling of risk: “My experience … was very, very difficult … [the most difficult] I ever had in my life. Entering those patients’ rooms, I’m fully geared but [it’s] terrifying and scary, feels like those little monster Coronas are everywhere.”

Contributing to the harsh work environment was uncertainty. A sonographer stated, “I think it’s still a guessing game, and I know everyone is doing their best, but it just makes it frightening.” Limited test supplies made it difficult to know for certain if patients and colleagues were COVID positive or negative. PPE was rationed for only verified COVID positive situations, leaving healthcare workers fearing they would contract COVID from undiagnosed patients or colleagues. After describing rapidly changing protocols, a physician captured the uncertainty by stating:

Our confidence in testing was shook when rumors began of patients initially tested negative, then later on testing positive. Then there was talk of patients presenting to the ER with atypical symptoms … There was even talk of asymptomatic patients presenting for other reasons such as a car accident and incidentally discovering on CT or chest x-ray imaging evidence of viral pneumonia with a subsequent testing positive. This was highly concerning to us because we were told not to wear a mask in the clean area so as not to panic people.

People also were voluntarily or involuntarily assigned to new units or put into new roles. “I, just like many people, got redeployed, moving from, forced actually, to be moved from their [outpatient] office to go and work directly with the COVID patients [in the hospital]”, voiced a medical assistant. A young nurse explained, “ … my unit was one of the first to convert to strictly Coronavirus patients. This really, really freaked me out, I was really unsure of what to expect. Mind you, I haven’t been a nurse for very long, so I was definitely scared about that.”

Perhaps most pervasive was a fear of inadvertently passing the virus to others, especially to one’s family. “The first few weeks I was pretty much a zombie,” noted a participant who was in an administrative support role. She continued, “I went into work every day … terrified for my family that I may bring something home. I would pretty much strip in the garage, spray everything down with Lysol, run to the shower and decontaminate myself … every night before I would even see my family.” The psychological distress of putting one’s family at risk was widely shared. A medical assistant described, “Every time you go in, you are risking your life and your family’s. … The feeling that I may get my family infected by this is just killing me…I can’t stop [thinking], what if somebody got sick? … I’m trying to do my job, then I put my family and myself at risk.”

Many of these fears were amplified when colleagues started to get diagnosed with COVID. “On top of this, many nurses, residents and physicians became ill making morale even lower,” said a physician, “One of the night nurses on a floor I worked on ended up critically ill, intubated, on dialysis, requiring ECMO, and was airlifted to an outside hospital. This weighed very heavy on the nursing staff … ”

Beyond the fear of contagion for self and family, healthcare workers were also distressed from working with very ill and dying patients, either for the first time or with increased frequency. A nurse described the challenges of shifting from a low acuity unit to one full of COVID patients, “There’s a lot of emotional trauma when caring for these patients … I’m seeing things that I never would have seen on my regular unit. I’m getting a lot of DNR patients … I think I’ve had 4 patients of mine pass away. You know, you never forget your first … it was very hard for me.”

Compartmentalizing these work-related stressors was impossible for many. One nurse stated “I feel like I’m not really enjoying my days off because all I do is think about work, think about the families of the patients that I have or am caring for. And it really weighs heavy on my mind.” Sleep disruptions (both insomnia and nightmares) were also reported. As a nursing assistant vividly described, “I felt so stressed out and so depressed and I still have difficulty sleeping. And if I do sleep it will be for only a few hours and I wake up, endless nightmares. The noise, the sounds of those ventilator breathing machines, the sounds of the patient’s breathing are still ringing in my ears.” She went on to describe a loss of appetite: “I can’t even eat at the hospital, I can’t even drink, to this day I cannot drink water. Everything tastes bad in my mouth. … I can’t go back to my normal routine to get a good meal. It just because of what I’ve seen, what I experienced.”

There was also distress around the stability and economic viability of different medical practices and, in turn, job security (something undoubtedly amplified by significant layoffs during the first surge). In particular, the disruption of less urgent, non-COVID patient services, as well as the fear and anxiety surrounding exposure, lead to staff loss and negative economic impact. One physician summarized these latent impacts of the pandemic:

This virus has ruined a lot of businesses including medical practices. In my practice we … basically had to go down to less than 5% during this pandemic and this lasted for over 3 months, so basically it bankrupted the practice. Staff became hysterical about many things and very paranoid about their work or coming to work. Many of them took off and did not ever come back…

Relatedly, those working in service lines that were taken down during the first surge felt they were not able to care for their patients. One office administrator articulated this, saying, “[I’m] very, very disappointed that patients have been cancelled [or] rescheduled for exams that they really need. [It] makes me feel guilty that we can’t really help patients out.”

A medical assistant who was redeployed from an office to the hospital left 2 recordings voicing frustration that the institution wasn’t doing enough for employees, “We did our job, 1000 people did their job, but we were not feeling like we were appreciated by the company … .”

In contrast to the severe working conditions and psychological distress described above, most participants simultaneously described rewarding aspects of the experience, including a sense of pride that came with fulfilment of their duties as healthcare workers. After describing a long process of feeling helpless and learning what was helpful and unhelpful to COVID positive patients, a participant stated, “I must say I’ve never felt more proud to be a physician in my life than when the nurse and I walked into a COVID positive room, looked the patient in the eyes, and said ‘we will take care of you’. In that minute all the fear melted away and there was a human being who needed us.” Another participant in an administrative/clerical support role voiced commitment after being told by physician colleagues how appreciative they were for her support, “ … And that really helped me, you know it really made me feel part of the team and it made me want to continue to be there and to help them, help everybody else.”

In addition to the support and pride derived from their colleagues, patients often served as a source of confirmation of purpose. As a nurse recounted, “My second shift that was so long, one of my patients was extubated, but she was doing so well, we chatted. And when I sent her to a progressive unit, she held my gloved hand and gave me a thumbs up. She filled me with adrenaline and made me confident this was a disease that could be beat.”

Finally, amidst the anxiety, exhaustion, and recognition of the lasting impact that the pandemic will have on the healthcare system, several participants also reported a focus on hope and optimism about the future, particularly as the epidemiological picture began to improve. One clerical staff member stated,

You just want to move on and then you start seeing the numbers drop and you start seeing floors go back to [being] normal surgical floors or … normal ICU floors versus COVID floors. And you start feeling so much better because you can see the light at the end of the tunnel… it feels really good to start seeing and feeling a little normalcy in this place. The world will never be the same place, things are always going to be different. We could see changes that probably will continue to take place. Video calls to doctors versus going in to see them.

### Theme 2: despite extreme isolation, healthcare workers formed intense and meaningful interpersonal connections in new ways.

Social isolation was described by most of the healthcare workers who participated in this project. For some, this was the worst part of working during the pandemic. As a nurse put it, “ … maybe one of the worst symptoms of the whole morphology of COVID is how isolating it is … . And I wonder how that’s all going to piece back tougher or if it can.” She went on to capture it metaphorically: “So I’m just kind of an island at the moment, a COVID island.”

While pervasive, the social isolation was experienced in a variety of different ways. Some described being isolated from colleagues due to redeployment, social distancing and wearing PPE. “You know the hospital, the hustle and bustle of a regular day hospital, what 3–4 months ago even, you know people in the hallways, laughing, joking to watch the mood go from that type of setting to graveyard quiet [was startling],” a clerical support staff remarked. A medical assistant described a similar disconnection and inter-collegial silence:

“ … then as my coworkers started to get deployed to other locations, to the hospitals, I started to worry even more. I didn’t get deployed; I was one to stay at my location. But it’s just been a constant worry about my friends, my coworkers, who mean a lot to me … you know it was hard … I feel like a lot of people were silent. We weren’t talking to each other; we were kind of just going about our lives day by day without connecting. And that was hard, it’s still hard.’

Healthcare workers also described feeling physically and emotionally isolated from their family members when they were at home. A clerical staff member noted, “ … my husband, didn’t want me to go to [my current] department. And I did and [COVID] hit, which just made the situation 100 times worse. I couldn’t tell him what I had to do every day because it, it would not have been good at all. The few times that I did try to have a conversation about what was going on, it got ugly. I just feel very alone … ” A nurse described the attendant uncertainties of having to move out of their home altogether to protect family members: “So with all of the stress that I was experiencing, I all of a sudden had to move out for who knows how long.”

Several healthcare workers spoke of their struggles trying to communicate and connect with their patients when physically distanced or covered by PPE. As one medical assistant put it:

You know it’s hard to connect with patients when they can’t see you. They can’t see your expressions, especially working with young children. I know that they’re fearful, and it’s sad to know that I’m making them scared instead of happy. They can’t see my smile, which is sad because I do love seeing my patients and I love connecting with them. And right now, it just feels hard. It feels like such a disconnect from human contact.

Others described the emotional and perhaps even moral distress of watching very ill patients suffer alone, isolated from staff and their own families. One nurse vividly recalled, “[There was] this lady, lying by herself, behind a closed wooden door … in the basement of the hospital, so no windows to look outside. This woman, this mother, this wife … friend, daughter, by herself behind a closed door, quite possibly dying.”

Yet while social isolation was clearly difficult, many participants connected with others in new ways. Amidst visitation restrictions, healthcare workers served as intermediators between ill patients and their families and often were patients’ only human connection. This led to intimate interactions and intensely meaningful interpersonal connections. One nurse put both experiences succinctly, “The next day we terminally weaned her. I held her hand as she died. I called her husband and her children and made sure they knew she wasn’t alone when she passed.” Another nurse described the way in which the situation gave her entry to conversations normally held by families in private:

It was just me and Ms. L. It was so lonely, so isolating, to be in those rooms. ‘Your husband wants to talk to you Ms. L.’ Her eyes opened up, she seemed to be coming around. … I held a phone to Ms. L’s ear … She responded to his questions about her last wishes. I just stood there holding the phone off to the side in my full-on PPE garb, like it was from a movie. My face behind a restrictive mask and face shield, isolated in my PPE. It crossed my mind that this was likely the last time they would speak to each other. Ms. L said, ‘Tell everyone I love them.’ That was it. The tears rolled down my face. I couldn’t even wipe them. I didn’t want to lose it; I had to be Ms. L’s nurse, be there for her if she needed. We hung up [and] I found her hand and gently held it. ‘Are you okay?’ She said, ‘Yes. Thank you for calling my husband’. She said it the rest of the shift.

Similarly, despite feeling disconnected from their colleagues due to redeployment, many participants appreciated the teamwork that developed during this difficult time, which paradoxically made them feel, in many ways, more connected to their colleagues. Several remarked that pulling together amidst the distress was a poignant feature of the experience. “But we’ve survived, and we worked together as a team. And I appreciate each of my co-workers because we got through it together, the nurses and the doctors and all of us. We survived and that’s how I remember it, working together.” An administrative assistant remarked, “So, I have to say the one thing that this whole situation or crisis has taught me, is that we really can band together as a family and help each other … ”

## Discussion

A number of studies have reported a variety of hardships experienced by frontline healthcare workers during the COVID-19 pandemic (Danet Danet, 2021; De Kock et al., 2021; Muller et al., 2020;). This study draws attention to the paradoxical impact of the COVID-19 pandemic on healthcare workers. While the negative toll has been intense and pervasive, most of the healthcare workers in our study also described incredibly important, meaningful, and rewarding experiences. In addition, this study highlights that healthcare workers felt both disconnected from their previous communities and yet intensely connected in new ways with their colleagues, patients, and patients’ families.

The COVID-19 pandemic has posed unique challenges to healthcare workers. Our participants described unimaginable surreal and terrifying working conditions. Consistent with other studies, the traumatic struggles noted by our study participants were attributed to uncertainty (caused by new roles and responsibilities; emerging protocols; and uncertainties about who was infected, how to treat the disease and how to prevent its spread), limited PPE, risk to self and family members, working with very ill and dying patients, and the disruption to the healthcare needs of non-COVID patients (Bennett, Noble, et al., 2020). Likewise, the resulting psychological distress experienced by our participants has been well-documented in other studies (Danet Danet, 2021; Q. Liu et al., 2020; Shreffler et al., 2020).

The degree to which our participants paradoxically and spontaneously expressed rewarding and meaningful experiences both across and within stories is intriguing and less represented in the literature. Many of our participants described feelings of pride centred around being able to help patients and their colleagues. Their ability to make a difference in people’s lives led to a sense of meaning, hope and optimism. Although not as pervasive and across both domains of meaningfulness and increased connectedness, a few other qualitative studies have also found some positive experiences by healthcare workers during the pandemic. For example, positive aspects of inter-professional teamwork and solidarity was reported among ICU nurses in Spain (Fernández-Castillo et al., 2021). Similarly, a study from China described altruism and greater team solidarity amongst nurses whom they interviewed during the pandemic. Their participants described psychological growth, increased professional identity and pride, with simultaneous positive and negative emotions (Sun et al., 2020). In another study, healthcare workers in Denmark described increased pride and wanting to be part of something bigger that created a balance with the COVID-related distress they were experiencing (Marsaa et al., 2021).

Research shows that meaningfulness is important for good mental health and that it mitigates negative outcomes in times of crisis (Schnell & Krampe, 2020). This has been the case during the COVID-19 pandemic as well. Meaning-focused coping strategies predicted better psychological adjustment in a national sample of Americans early in the pandemic (Park et al., 2021). Similarly, meaningfulness was negatively predictive of depression and anxiety in a study of the general population in Turkey (Korkmaz & Güloğlu, 2021) and was found to be a buffer of COVID stress and general mental distress during the pandemic in Austria and Germany (Schnell & Krampe, 2020). Finding ways to help healthcare workers maintain a sense of meaning, value and pride during challenging times is warranted.

The importance of human connection was apparent in the second unifying theme that emerged from our data: Despite extreme isolation, healthcare workers experienced intense and meaningful interpersonal connections in new ways. The feelings of isolation and disruption to normal ways of connecting to patients, colleagues and their own families was devastating to our participants, similar to those reported in other studies. For some of the participants in our study, it was the worst part of working during the pandemic. For several participants though, this disruption in normal interpersonal interactions paradoxically resulted in new, and in many ways, more intense connections with others. While lack of connection resulted in a great deal of distress, those who described more intensely intimate interactions with their patients and enhanced teamwork with their colleagues found these new interactions profoundly rewarding. The impact of social connectedness and social support on overall health and well-being is well established in the literature (Donovan & Blazer, 2020; Prakash et al., 2020; Saltzman et al., 2020). Recent reports demonstrate the role that social connectedness can play in buffering against negative sequalae experienced by healthcare workers during the COVID-19 pandemic (Fernández-Castillo et al., 2021; Labrague & De Los Santos, 2020; Nelson et al., 2021; Nitschke et al., 2021; Yıldırım et al., 2021). For example, Yildirim et al reported that healthcare workers in Turkey with higher levels of coronavirus stress experienced less optimism and social connectedness which contributed to higher COVID-19 related burnout levels (Yıldırım et al., 2021). Conversely, in a study from the Philippines, COVID-19 front-line nurses who reported higher levels of perceived social support reported lower levels of anxiety (Labrague & De Los Santos, 2020). Our study adds to this literature to suggest that increased social connectedness is occurring even amidst simultaneous feelings of social isolation, and perhaps can be leveraged to the benefit of healthcare workers’ mental health.

This qualitative study was unique in its approach of using web technology to collect unstructured, anonymous audio recordings. To our knowledge, only one other qualitative study of healthcare workers during the pandemic used an audio diary approach (Bennett, Noble, et al., 2020). Bennet, et al. also noted that some healthcare workers in their study expressed positive experiences, although no consistent positive themes emerged from their data. Conversely, they reported a theme that did not emerge as a theme in our data. In their analysis anger towards the health system was a salient theme, something that a few of our participants mentioned but not enough to emerge as a theme from our data. The differences in our findings may be due to differing location and culture of the health systems, different recruitment strategies (they recruited via twitter), and different timing of data collection.

Participants clearly took advantage of the flexibility of our web-based platform, sharing their thoughts at times that would not have occurred if we relied on investigator led interviews. Four of the recordings occurred between 1:50 am and 4:20 am three occurred between 6:20 am and 7:52 am. They participated in moments that were either most convenient for them or when they felt the urge to share their stories. It is likely that this flexibility extended our reach to people who would not have participated in a study at a scheduled time. Participants could choose to participate while on break at work or when they were home. We suspect that this gave voice to more night-shift workers as a result.

Others have reported that audio diaries provide opportunities for more depth and reflection of experiences (Markham & Couldry, 2007). We believe that the degree to which our participants described unprompted paradoxical experiences and the richness of their stories was at least in part because they had time to reflect at their own pace on what they wanted to share. Based on the intonation of one participant’s narrative, it was clear she was reading a story she had taken the time to write out, ensuring she captured everything she wanted to say. Her story ended right at the five-minute cut-off.

Web-based audio diaries have the potential to capture narratives from very large sample sizes due to ease of access that is not dependent on interviewer availability and scheduling. However, participation was surprisingly low in our study. This may have been due to several technical hurdles that ended up making our system somewhat difficult to access. For example, the default browser used in the health system was not supported by the third-party audio recording application, which necessitated an additional step for participants to cut and paste the link to a supported browser. Additionally, there were multiple recordings that did not get properly saved, which may have been due to user error or technical glitches. Now that we have addressed various technical hurdles, we anticipate increased participation rates next time we use this platform. It is also possible that leaving a verbal recording was intimidating for some participants. Researchers using this technology in the future may wish to consider allowing participants to choose between leaving audio stories or uploading written stories. Finally, alternative recruitment strategies should be considered in future research. Our use of invitations periodically embedded in corporate emails may not have reached as many people as we had hoped. In addition, some may have confused this research study with other attempts by the health system to collect feedback from its employees.

Another expectation was that the audio recording tool would provide an outlet for some healthcare workers who were experiencing isolation due to the nature of their work (Bennett, Hunter, et al., 2020) with the added benefit of sense-making, alleviating discomfort from the experience, and facilitating coping with stressors (Pennebaker & Seagal, 1999). Although we did not directly assess this outcome and this theme did not emerge in our analysis, one participant who submitted multiple recordings noted that she lived alone, was redeployed with colleagues she didn’t know, and didn’t have anyone to talk to. She was concerned that she was overusing the study site. Another participant thanked us for the opportunity to share her story. This is consistent with the potential therapeutic value that others describe from the use of audio-diaries (Bennett, Hunter, et al., 2020; Pennebaker & Seagal, 1999).

Audio diaries have an advantage over other qualitative data collection strategies in that they minimize the influence of the researchers on the participant’s responses (Monrouxe, 2009). We agree that this may lead to more accurate accounts of what is on participants’ minds without censorship based on what is perceived as socially desirable responses. However, we also acknowledge that there is a flipside to this. Without an interviewer present, there are no opportunities to ask follow-up questions to clarify what the participant meant and to seek more detail for a potentially deeper understanding of what the participant is trying to communicate. This potential disadvantage should be weighed against the many advantages that a web-based, audio diary platform provides.

*Limitations*: This study’s limitations included a relatively small sample size that may represent only people who had particularly distressing or rewarding experiences. It is possible that our study was biased for healthcare workers who were coping well and had the fortitude to submit a personal story. It may have also been biased towards healthcare workers who were more technologically savvy and who regularly used their work email. Only about ten percent of the people who completed demographic information followed through to leave a recorded story. This may have been partly due to the technical issues or potentially intimidating nature of leaving an audio recording described above.

The timing of our study may have influenced both participation and the content of participant stories. We started collecting data while healthcare workers were still at the peak of the first surge, still in the midst of chaos, and filled with physical and emotional exhaustion. This may have felt like one more thing they were being asked to do and may have occurred too close to the traumatic experience they had been going through. Based on the date stamp and sequence of the stories, the paradoxes of both positive and negative experiences occurred when still at the peak of the surge and as it neared the bottom.

## Conclusion

COVID-19 created an intensely harsh and traumatic work environment for healthcare workers. Paradoxically, amid social isolation and extreme distress, a sense of value and meaning and new ways of forming human connections naturally emerged. Our results suggest that responding to distress and burnout among healthcare workers should rely as much on enhancing and leveraging positive experiences as mitigating negative ones. For example, amplifying the feelings of teamwork, solidarity, and sense of purpose may be as effective as offering outlets for debrief and counselling about distressing experiences. Such strength-based interventions may be particularly useful to address the evolving challenges as the pandemic continues.

The audio diary approach used in our study created a space that allowed participants to reflect deeper on their experiences which likely contributed to our paradoxical findings. Further work is needed to determine how to enhance participation given the potential this methodology has in reaching large populations. Anonymous, web-based platforms are a unique way to collect qualitative data and should be considered when investigating new and complex situations such as the one posed by COVID-19.

## Supplementary Material

Supplemental Material

## Data Availability

Data are available on reasonable request. Raw transcripts of the data analysed in the study are available from the lead author.
