# Supplementary material for: Paradoxical experiences of healthcare workers during COVID-19: a qualitative analysis of anonymous, web-based, audio narratives
Source: Int J Qual Stud Health Well-being. 2023 Mar 2;18(1):2184034. doi: 10.1080/17482631.2023.2184034 (PMC9987722; doi:10.1080/17482631.2023.2184034)
Supplement: Supplemental Material [file ZQHW_A_2184034_SM4056.zip › Supplementary files/Supplemental Figure 1.docx]

**Supplemental Figure 1: Data flow diagram.**
